# Photodynamic Therapy Modulates pri-miRNA Expression in *C. albicans*-Infected HEK-293 Cells: An In Vitro Study

**DOI:** 10.3390/cimb47110949

**Published:** 2025-11-14

**Authors:** Cinzia Casu, Andrea Butera, Alessandra Scano, Andrea Scribante, Valentino Natoli, Mara Pinna, Sara Fais, Germano Orrù

**Affiliations:** 1 Oral Biotechnology Laboratory, Department of Surgical Science, University of Cagliari, 09124 Cagliari, Italy; cinzia.casu2@unica.it (C.C.); alessandra.scano77@unica.it (A.S.); mme.pinna@gmail.com (M.P.); sara.fais@unica.it (S.F.); orru@unica.it (G.O.); 2 Unit of Dental Hygiene, Section of Dentistry, Department of Clinical, Surgical, Diagnostic and Pediatric Sciences, University of Pavia, 27100 Pavia, Italy; andrea.scribante@unipv.it; 3 Unit of Orthodontics and Pediatric Dentistry, Section of Dentistry, Department of Clinical, Surgical, Diagnostic and Paediatric Sciences, University of Pavia, 27100 Pavia, Italy; 4 Department of Dentistry, School of Biomedical and Health Sciences, European University of Madrid, 28670 Madrid, Spain; 5 Azienda Ospedaliero Universitaria di Cagliari, AOU Cagliari, 09124 Cagliari, Italy

**Keywords:** pri-miRNAs, *Candida* spp., *C. albicans*, Photodynamic Therapy, *C. albicans* CA97

## Abstract

Oral infections caused by *Candida* spp. represent a major health concern due to the increasing resistance of these fungi to conventional antifungal agents. Photodynamic therapy (PDT) is a treatment based on the use of light at a specific wavelength that activates a photosensitizer (PS) in the presence of oxygen. The activated PS selectively binds to infected cells and induces apoptosis through the generation of reactive oxygen species (ROS). Previous biomolecular studies on *Candida albicans* have demonstrated that its infection triggers characteristic molecular signals, such as miRNA-146a and miRNA-155, which serve as inflammatory markers. This in vitro study aimed to evaluate the impact of PDT on the expression of their primary transcripts (pri-miRNAs) in a cell culture model of *C. albicans* infection. Human embryonic kidney (HEK-293) cells were infected with a multidrug-resistant strain of *C. albicans* (CA97) and subsequently exposed to curcumin-based PDT activated by blue light (470 nm). The expression of pri-miRNAs 146a and 155 was assessed before and after PDT treatment for each experimental group. The expression levels of pri-miRNAs increased approximately 2- to 3.5-fold following *C. albicans* infection but returned to baseline values after PDT treatment. The evaluation of pri-miRNAs 146a/155 may serve as a valuable research tool for monitoring early inflammatory responses induced by Candida infection, as well as a sensitive biomarker for assessing the effectiveness of photodynamic therapy in an in vitro cell culture model.

## 1. Introduction

Oral candidiasis is one of the most common fungal infections of the oral cavity. It is primarily caused by *Candida* spp., with *Candida albicans* being the most prevalent species; however, other species such as *C. glabrata*, *C. krusei*, and *C. parapsilosis* are increasingly reported. *Candida* spp. are commensal organisms present in more than 50% of healthy individuals [1,2]. Oral candidiasis is generally more frequent in elderly patients than in younger individuals [1]. Infection occurs when there is an imbalance between host immune defenses and fungal virulence. In vivo, the initial signs of *C. albicans* infection are typically represented by clinical manifestations, and currently, there are no established predictive biological markers. In vitro, the infection can be modeled using cultured cells and evaluated with various molecular markers, as previously described by different authors [1,3].

Clinically, oral candidiasis can be classified as acute or chronic, and as primary or associated forms. Primary forms include pseudomembranous candidiasis—the classic form characterized by removable whitish membranes; erythematous candidiasis, including subprosthetic forms (the most common type); atrophic candidiasis, primarily affecting the dorsum of the tongue; and chronic hyperplastic candidiasis, which commonly affects the buccal mucosa near the mouth corners and carries a malignant transformation risk of approximately 12% [1,3,4]. Associated forms include angular cheilitis, often co-infected with *Staphylococcus aureus*; median rhomboid glossitis, characterized by papilla loss on the tongue dorsum and susceptibility to fungal superinfection; and linear gingival erythema, a non-plaque-related gingival redness [1,2,3].

The conventional treatment of oral candidiasis is pharmacological, relying mainly on topical polyenes and azoles such as nystatin (100,000 IU), clotrimazole, or miconazole 1% [5]. However, increasing resistance to both topical and systemic antifungals—especially among multidrug-resistant (MDR) species like *C. krusei*—has prompted the exploration of alternative therapeutic approaches, including probiotics, ozone therapy, and photodynamic therapy (PDT) [6,7].

Photodynamic therapy is an emerging antimicrobial strategy involving three key elements: a photosensitizer (PS) that binds to microbial molecules, a light source (laser or LED) at a specific wavelength that activates the PS, and the presence of oxygen. When these components interact synergistically, they generate singlet oxygen and other ROS that selectively destroy microbial cells while remaining safe for host tissues [8,9]. Both in vitro and in vivo studies have demonstrated the efficacy of PDT against *Candida* spp., although experimental protocols are often highly heterogeneous [10,11,12]. The use of natural photosensitizers—such as curcumin, riboflavin (activated by 440–480 nm light), plant-derived compounds [13,14], and, more recently, lactoferrin (activated by violet light) [15]—is gaining increasing attention.

Diagnostic approaches for oral infections increasingly emphasize non-invasive methods that assess not only fungal load but also the host response and the cellular impact of infection [16]. Among these host-derived biomarkers, microRNAs (miRNAs) are small, non-coding RNA molecules (approximately 20–22 nucleotides) that mediate post-transcriptional gene regulation and are released from cells via lipid-encapsulated vesicles [17].

The biogenesis of miRNAs begins with the transcription of primary transcripts (pri-miRNAs) by RNA polymerase II [18,19]. These precursors, typically several kilobases long, contain a characteristic stem–loop structure comprising a double-stranded stem region and an apical loop [20]. Following nuclear processing, pre-miRNAs are exported to the cytoplasm, where they are further processed, incorporated into argonaute complexes, and can ultimately be secreted as mature miRNAs via exosomes—small vesicles mainly composed of lipids and proteins [20].

Each disease type is associated with distinct miRNA expression patterns, but the full range of signals released by infected cells remains incompletely understood. Because miRNAs often appear prior to clinical symptoms, they may serve as early biomarkers of infection or inflammation [16,17,18,19]. Several studies have correlated oral diseases with miRNA expression, including oral cancer [21]. Some of the miRNAs most frequently linked to inflammation are miR-146a and miR-155 [22,23].

Emerging evidence indicates that miR-146a and miR-155 play crucial roles in regulating pathophysiological processes during fungal infections, including apoptosis, macrophage recruitment, and proinflammatory cytokine production [24]. Furthermore, miRNA expression can reflect treatment efficacy, drug responsiveness, and patient prognosis [22,23,24].

The present study aimed to quantify the expression of pri-miRNAs 146a and 155 in HEK-293 cells infected with *C. albicans* and subsequently treated with curcumin + H_2_O_2_-based PDT. We evaluated the effects of PDT on the modulation of inflammatory molecular signaling and assessed whether pri-miRNA expression could serve as a diagnostic and prognostic indicator of treatment efficacy.

## 2. Materials and Methods

### 2.1. Cell Cultures and Infection

Human embryonic kidney (HEK-293) cells were used for this in vitro study (see paragraph 7 in Section 4). Cells were cultured in 6-well plates (Sigma Aldrich, Milan, Italy) using Minimal Essential Medium (MEM) supplemented with 10% fetal bovine serum (FBS) and 1% penicillin/streptomycin (100 IU/mL; 100 μg/mL). The cells were maintained in flasks until a density of 1 × 10^6^ cells/mL was reached (after five passages). For each experimental condition, 1 × 10^4^ cells were seeded per well.

**Figure 1:**
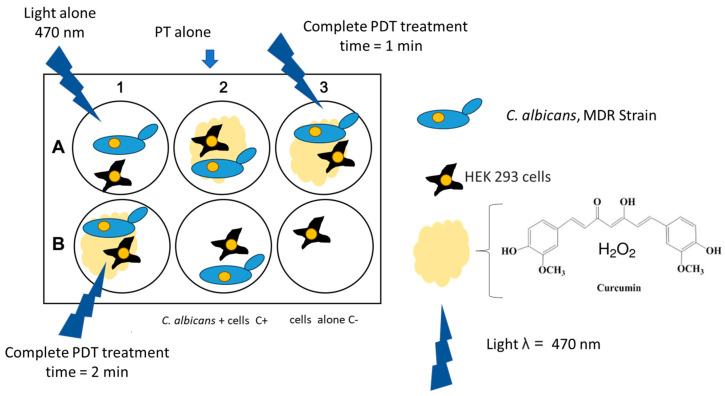
Schematic representation of the in vitro experiment.

The cells were incubated in a humidified atmosphere of 5% CO_2_ at 37 °C until confluence (approximately 48 h). At this stage, the cells were infected with a suspension of a clinical multidrug-resistant (MDR) isolate of *Candida albicans* (CA97) at a multiplicity of infection (MOI) of 1:1. The experimental design is shown in Figure 1 and all experiments were performed in biological triplicate. After 12 h of incubation, PDT treatment was applied. RNA extraction was performed 1 h after PDT using a spin RNA extraction kit (Anatolia, Istanbul, Turkey) according to the manufacturer’s instructions.

Although the selected cell line is of renal origin and not epithelial, this model was adopted as a preliminary system to study *C. albicans*–human cell interactions [25]. This approach was based on previous studies comparing fungal infections across different cell types. Furthermore, HEK-293 cells are commonly used to evaluate the toxicity of various natural compounds, including curcumin [26].

### 2.2. Photodynamic Therapy Process

The light source used for PDT was a custom-built LED lamp emitting blue light at a wavelength of 470 nm, with a power output of 200 mW, a 5 mm light spot, and a fluence of 10,204 W/cm^2^. This laboratory-made device was employed to avoid *Candida* spp. contamination of commercial PDT equipment.

The photosensitizer (PS) consisted of a mixture of curcumin and 3% hydrogen peroxide (H_2_O_2_). For each culture well, 0.5 mL of the PS solution was applied. This formulation reproduces a commercial photosensitizer (Curcumin + H_2_O_2_, CMS Dental, Copenhagen, Denmark) previously validated in the literature [27,28]. The rationale behind this combination is that a small amount of H_2_O_2_ acts as a catalyst, providing additional oxygen and reactive oxygen species immediately available to initiate the photodynamic reaction.

**Figure 2:**
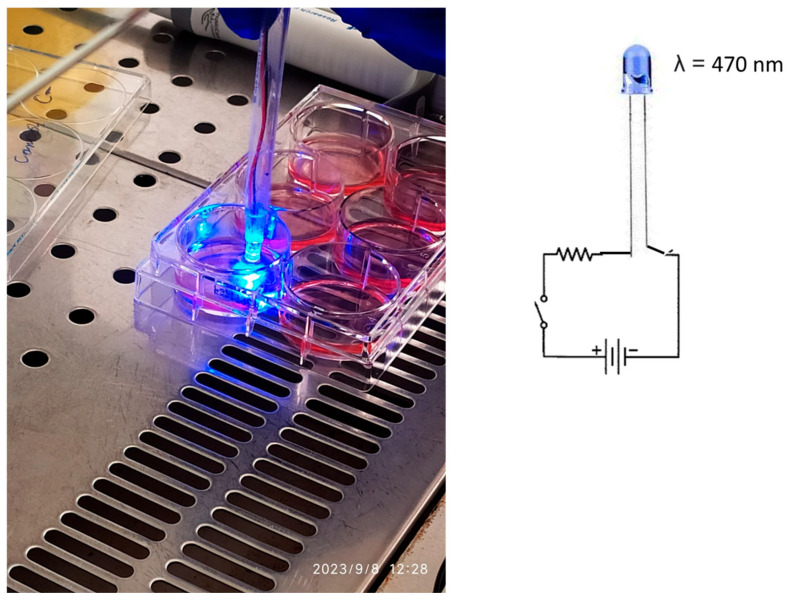
The cell HEK-293 (human embryonic kidney) culture, and the lab-made device used to emit light at 470 nm radiation.

After applying the PS to the infected cell surface, the LED lamp was positioned at a distance of 1 cm and activated for 1 or 2 min. Figure 2 illustrates the PDT procedure performed on the infected HEK-293 cell cultures.

### 2.3. Experimental Groups

The experiment was conducted in triplicate using 6-well tissue culture plates. Each plate included six experimental groups: (i) uninfected and untreated cells (negative control); (ii) infected cells without treatment; (iii) infected cells exposed only to the photosensitizer; (iv) infected cells exposed only to blue light; and (v) infected cells subjected to the complete PDT protocol (photosensitizer + LED light) for 2 min.

### 2.4. Measurement of Pri-miRNAs

Forward and reverse primers were designed based on genome sequences with accession numbers MN298232.1 (pri-miRNA-146a) and NC_000021.9 (pri-miRNA-155) (Table 1). Primer optimization and normalization were initially performed in silico using the Oligo 6 software (MedProbe, Oslo, Norway) and the DINAMelt server, which simulates nucleic acid melting profiles as a function of temperature.

Hybridization parameters were set as follows: monovalent cation concentration = 0.05 mol/L, Mg^2+^ = 0.002 mol/L, probe/target concentration = 100 mM, and initial hybridization temperature = 37 °C. The secondary structure of PCR amplicons, oligonucleotides, and pri-miRNAs was predicted using the UnaFold program (https://www.unafold.org/help.php, accessed on 21 July 2025) with the same parameters.

The miRNAs were quantified by relative quantification (2^–∆∆Ct^ method) by using beta-actin as a reference housekeeping gene; in this case, the oligos used are OG650 (beta-act) F 5′-GCATGGGTCAGAAGG-3′ and OG651 (beta-act) R 5′-AGGCGTACAGGGATAG-3′. The real-time PCR conditions for amplifying the reference gene were the same as described below for miRNA (link-8).

### 2.5. Real-Time RT-PCR Conditions

SYBR^®^ Green real-time PCR was performed using the TaqPath™ 1-Step RT-qPCR Master Mix (Life Technologies, St. Louis, MO, USA). Each 20 μL reaction contained 5 μL of master mix, 2 μL of SYBR^®^ Green solution (1:1000 dilution; Sigma-Aldrich, St. Louis, MO, USA), 1 μL of each primer, 5 μL of RNA extract, and 7 μL of nuclease-free water.

Amplification was carried out on a CFX96™ system (Bio-Rad, USA) with the following thermal profile: (i) uracil-DNA-glycosylase (UNG) incubation at 25 °C for 2 min; (ii) reverse transcription at 50 °C for 15 min; and (iii) 40 cycles of 95 °C for 3 s, 60 °C for 30 s, and 81 °C for 2 s. Fluorescence was measured at the end of each 81 °C step.

All experiments were performed in triplicate. The standard deviation (SD) of the threshold cycle (Ct) values ranged within ±0.8 Ct. The secondary structures of pri-miRNA amplicons were also evaluated using the UNAFold Web Server (https://www.unafold.org/ accessed on 21 July 2025).

For each plate, pri-miRNA expression levels before and after treatment were compared, and differences were interpreted as effects of light exposure, photosensitizer action, or the full PDT procedure.

### 2.6. Antimicrobial Susceptibility Test

Both treated and untreated HEK-293 cell cultures were subjected to colony-forming unit (CFU) analysis to evaluate *Candida* viability. Briefly, 200 μL of culture medium was inoculated into a tube containing 15 mL of molten Sabouraud agar (Microbiol, Uta, Italy) at 50 °C. After thorough mixing, the medium was poured into 90 mm Petri dishes. Following solidification, colonies were counted after 24–48 h of incubation at 37 °C, and results were expressed as CFU/mL.

Antifungal susceptibility of the *C. albicans* CA97 strain to azoles was determined using sterile flat-bottom 96-well microplates, following the guidelines of the Clinical and Laboratory Standards Institute (CLSI) (https://clsi.org/shop/standards/m27/, accessed on 21 July 2025).

### 2.7. HECK-293 Cell Line

As a preliminary model, an immortalized HEK-293 cell line with epithelial-like morphology was employed. Compared with primary oral cells, HEK-293 cells are less susceptible to early senescence, which can significantly alter mRNA expression patterns during long-term experiments.

Previous studies have reported that miRNAs 155 and 146a are downregulated during cellular senescence, with time-dependent expression [28,29]. Because PDT represents a dynamic antimicrobial process requiring a stable cellular response, the use of an immortalized line minimizes age-related transcriptional variability. Thus, HEK-293 cells provide a consistent molecular target for evaluating different PDT protocols.

### 2.8. Statistical Analysis

All experiments were performed in triplicate (three biological replicates). Quantitative data were expressed as mean ± standard deviation (SD). Gene expression changes of less than 0.5-fold or greater than 2-fold were considered significant.

Each sample underwent three independent RT-PCR runs, performed in duplicate. Statistical significance among the experimental groups was assessed using one-way ANOVA via the online Social Statistics test tool (https://www.socscistatistics.com/tests/anova/default2.aspx, accessed on 21 July 2025).

## 3. Results

**Figure 3:**
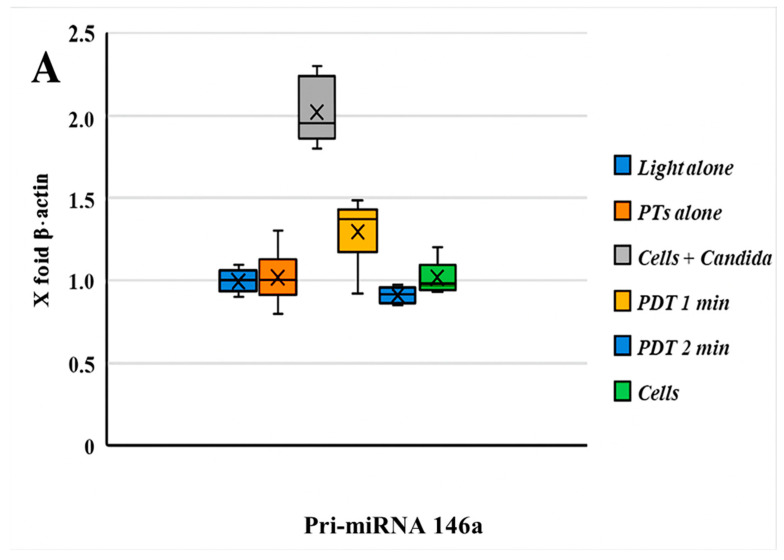

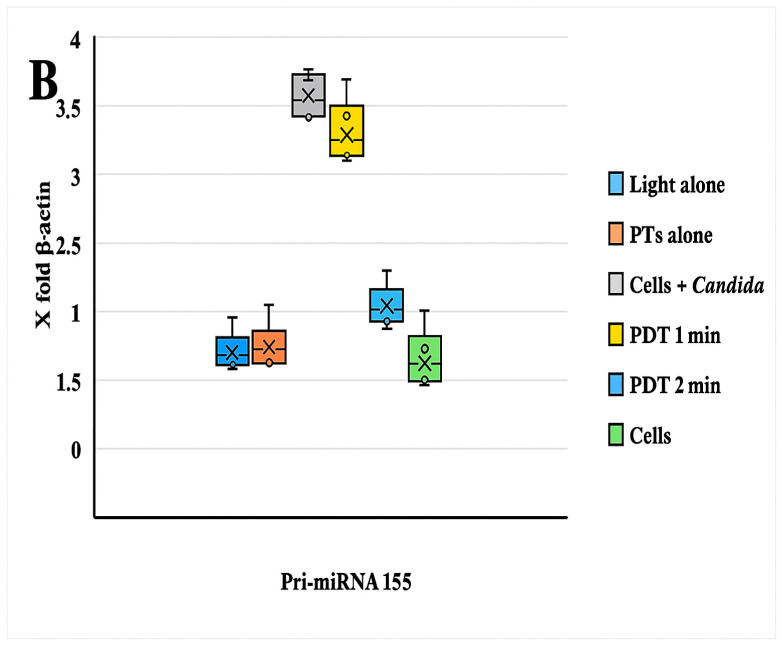
(**A**) The graph reported the values of the expression of Pri-miRNA 146a before and after PDT; comments are summarized below. (**B**) Boxplot graphs regarding the miRNA 155 expression patterns of HEK-293 cells infected with an MDR strain of *C. albicans* with/without PDT treatment and different combinations. A significant statistical difference between HEK-293 cells infected with *C. albicans* was observed only with PDT treatment after 2 min of light exposition (*p* < 0.05). A decrease in the pri-miRNA 146a and pri-miRNA 155 expression levels (compared with β-actin = 1) was observed during PDT with a light wavelength of 470 nm for 1 min.

**Figure 4:**
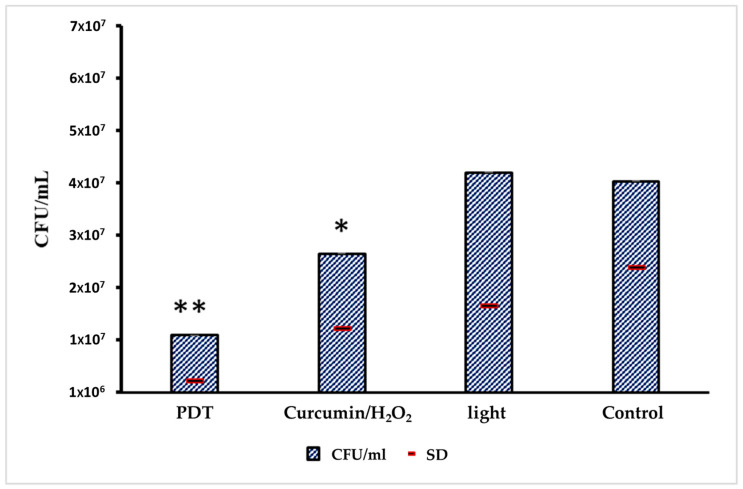
Evaluation of antimicrobial activity of the PDT on *C. albicans* cells inoculated in HEK-293 line cells. From the left to the right: CFU in the PDT group; CFUs in the group only treated with PS; CFUs only exposed to light; and CFUs of negative control (no treatment). The standard deviation values are represented by a red line; 3 technical triplicates were obtained from 3 biological triplicates; and a weighted average on different dilutions was used. Only the control in comparison with the PDT treatment showed a significant difference (** *p* < 0.05); the other combinations were not significant (* *p* > 0.05), using the ANOVA statistical approach.

The comparative expression levels of pri-miRNA 146a and pri-miRNA 155 before and after photodynamic therapy are presented in Figure 3. The results for Pri-miRNA 146a and Pri-miRNA 155 expression in HEK-293 (human embryonic kidney) cells are illustrated in Figure 3A,B. This experimental stage has reported that the entire photodynamic treatment was more effective in comparison with the photosensitizer or light alone. The relative expression levels of miRNA 146a and miRNA 155 decreased by about 50% after 2 min of treatment: from 2 to 0.9 for miRNA 146a and from 3,4 to 1,5 considering miRNA 155. Values were measured after 2 min of PDT irradiation. This in vitro assessment also showed that the decrease in miRNA processing is related to the 470 nm light exposition time. Figure 4 shows the antimicrobial activity of PTD on *C. albicans* cells.

*C. albicans* CFU reduction was observed in wells treated with complete PDT or with curcumin/H_2_O_2_ only. The CFU reduction with PDT was 74.7% in comparison with control growth, which aligns with our previous research indicating that PDT acts as a fungistatic agent against the MDR strain. By considering the drug susceptibility of this strain to azoles, a complete inhibition (minimum fungicidal concentration) was observed for fluconazole > 64 μg/mL, voriconazole > 8 μg/mL, and ketoconazole 4 μg/mL.

## 4. Discussion

This in vitro study demonstrated that photodynamic therapy (PDT) induces a decrease in the expression of pri-miRNAs 146a and 155 in *Candida albicans*-infected HEK-293 cells, with results varying according to the duration of illumination (1 or 2 min). 

Previous investigations evaluating the efficacy of PDT against *Candida* spp. primarily focused on colony-forming unit (CFU) counts [13,25] or zones of inhibition using the Kirby–Bauer method [15,26]. However, very few studies have examined the impact of PDT on infected mammalian cell cultures, rather than directly on fungal suspensions or biofilms [28,29]. To our knowledge, no previous reports have investigated miRNA or pri-miRNA expression in a fungal infection cell model following PDT exposure, making this study the first of its kind.

Although the most established PDT protocols for *Candida* spp. employ toluidine blue or methylene blue activated by red lasers or LEDs (630–660 nm) [27,30], the recent literature increasingly supports the use of natural photosensitizers, such as curcumin derived from *Curcuma longa* [29,31]. Curcumin is biocompatible, ingestible, and effective, often performing as well as—or better than—synthetic photosensitizers [27,30,31].

Moreover, the addition of hydrogen peroxide to curcumin, already validated through a commercial formulation, enhances the generation of reactive oxygen species (ROS) and improves antifungal efficacy against MDR *Candida* spp. when activated by blue (460 nm) or polarized light sources [29]. These findings provided the rationale for using this combination in the present study.

Although curcumin-based PDT has been extensively evaluated for its antifungal effects on biofilms, its influence on host cell signaling had not yet been explored. Our results show that in infected HEK-293 cells, PDT not only reduces fungal CFUs but also modulates inflammatory signaling pathways by downregulating pri-miRNAs 146a and 155.

The selection of the HEK-293 model is justified by its frequent use in biomedical research due to its high reproducibility and stability under in vitro conditions [32,33,34,35,36]. Despite originating from renal tissue, this cell line exhibits biochemical and physiological characteristics comparable to epithelial cells, making it suitable for infection and cytotoxicity studies.

Both miR-146a and miR-155 have been extensively validated as inflammatory markers in oral and systemic diseases [37,38]. Because mature miRNAs are often secreted via exosomes and can fluctuate depending on sampling timing, evaluating pri-miRNA precursors provides a more consistent and sensitive measure of intracellular transcriptional activity [39]. 

To date, pri-miRNAs have not been used as indicators of the efficacy of topical antimicrobial therapies. However, our findings suggest that they can reliably reflect cellular responses to PDT, offering a promising molecular tool for monitoring inflammation and treatment outcomes in vitro.

The observed decrease in pri-miRNA 146a and 155 expression following PDT highlights the therapy’s potential to modulate host inflammation triggered by fungal infection while confirming its direct antifungal action. The recorded 74.7% reduction in CFUs after PDT aligns with previous reports showing that curcumin-based PDT exerts a fungistatic rather than fungicidal effect [28].

The duration of light exposure appeared to significantly influence pri-miRNA downregulation, with greater effects observed after 2 min of illumination. Previous research has shown that ROS and singlet oxygen are generated within seconds of PDT activation [26,29,40]; therefore, the additional reduction in miRNA expression after prolonged exposure could be associated with secondary photothermal or oxidative stress effects, though these are minimal under our parameters (200 mW) [26,29,41]. Determining the optimal illumination protocol will be essential for defining effective and safe in vivo PDT applications.

The concept of evaluating tissue-derived pri-miRNAs as biomarkers, in addition to salivary or serum miRNAs, is intriguing because it could provide a reliable approach for assessing localized cellular responses, especially in tissues like the oral mucosa where salivary clearance may interfere with exosome-based measurements. This preliminary model can therefore serve as a foundation for future studies investigating the cellular response to topical pharmacological agents or device-based therapies such as PDT in bacterial, fungal, and viral infections.

The antimicrobial results shown in Figure 2 confirm a modest yet consistent antifungal effect. Although previous work indicated that curcumin-mediated PDT primarily exerts a bacteriostatic effect against *Candida* spp., the results of this study demonstrate its additional anti-inflammatory potential through miRNA modulation [42]

### 4.1. Interaction Between miRNAs and PDT Treatment

This work represents an innovative step toward integrating molecular biomarkers into the evaluation of PDT efficacy. Previous studies have reported that miRNAs are responsive to light-based therapies. For instance, Borgia et al. (2021) [43] reviewed the involvement of microRNAs in phototherapy and PDT, particularly in dermatological applications, where several miRNAs were shown to be either up- or downregulated after treatment. Among these, miRNA-155 consistently emerged as a diagnostic indicator of treatment effectiveness or resistance.

Similarly, Soonthornchai et al. [44] observed that miR-155 expression decreased following combined light and methotrexate therapy in psoriasis, likely through the activation of the caspase-3 apoptotic pathway. Additionally, miR-146a was found to mediate the reduction in inflammatory processes via the TRAF6/IRAK1 pathway, with both mechanisms closely associated with oxidative stress regulation.

In our model, pri-miRNA expression increased following *C. albicans* infection, consistent with Wei et al.’s findings (2019) [45], who demonstrated that infection elevates both proinflammatory cytokines and miR-155 levels through activation of the Dectin-1–Syk/Raf-1–MAPK signaling pathway [46]. They further proposed that miR-155 may act as a negative feedback regulator, limiting excessive cytokine release during infection [47,48].

Our findings suggest that PDT exerts a dual action—targeting both fungal cells and host cells. The rapid burst of ROS generated by PDT may trigger the antioxidant defense mechanisms of HEK-293 cells, leading to the downregulation of the pri-miRNAs involved in inflammation. Conversely, *C. albicans* cells, lacking an efficient antioxidant system, are more susceptible to oxidative damage, resulting in growth inhibition [49,50,51].

**Figure 5:**
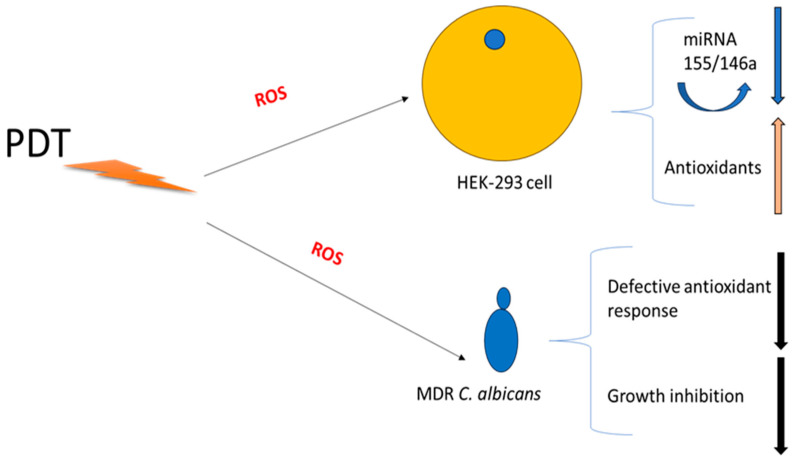
Schematic Hypotheses for dual effect of PDT.

Figure 5 illustrates a potential explanation for the dual effect of PDT treatment on host cells and Candida albicans. The fungal inhibition could be due to no performant antioxidant machinery in fungi in comparison with HEK-293 cells; downregulation of miRNAs 155 and 146a could be linked to this process. Another interesting mechanism of fungal killing and host cell miRNA production could be explained by caspase-3 and NF-kB pathways that are strictly linked to inflammasome, ROS production, and subsequent miRNA regulation. The mechanism is very intricate, but briefly, this multi-protein complex mediates the activation of caspase-1, which promotes the secretion of the proinflammatory cytokines IL-1β and IL-18 as well as pyroptosis, a form of cell death induced by bacterial pathogens, and understanding these pathways may provide insights into disease pathogenesis that might serve as potential targets for therapeutic intervention. (i) *Candida albicans* during mucosal infections involve the cooperative action of innate and adaptive immune effectors with regard to inflammasome activation in interleukin-releasing cytokines; (ii) PDT could occur early and hyperactivate the inflammasome by ROS production; and (iii) this activation is an inflammatory feedback pathway including caspase-3 and NF-kB. (iv)

### 4.2. Limitations of This Study

This investigation should be regarded as a preliminary study, given that it employed an immortalized HEK-293 cell line, which—although suitable for standardization—differs from primary oral epithelial cells. Future work should include experiments using primary gingival epithelial cells or oral keratinocytes to better reproduce in vivo conditions. Additional molecular assays evaluating oxidative stress-related genes (e.g., catalase, BAX, BCL-2, HO-1) and broader pri-miRNA profiles would help clarify the mechanisms underlying PDT’s molecular effects.

Another limitation is the small sample size and the exploratory nature of the experimental design, aimed primarily at validating the model before large-scale implementation. Although both PDT and miRNAs 146a/155 have been individually validated in vivo, to date, no studies have assessed their combined diagnostic and therapeutic potential. The present work therefore represents an essential first step toward integrating molecular biomarkers with PDT evaluation.

## 5. Conclusions

This study highlights two key findings. First, photodynamic therapy (PDT) demonstrated a significant ability to modulate inflammation in *Candida albicans*-infected cells by downregulating inflammatory pri-miRNAs (146a and 155). Second, the assessment of pri-miRNA expression proved to be a sensitive and non-invasive molecular tool for monitoring both the inflammatory status and the therapeutic outcome of PDT in vitro.

Together, these results suggest that PDT, particularly with curcumin + H_2_O_2_ activated by 470 nm blue light, represents a promising strategy not only for antifungal control but also for regulating host cellular responses to infection.

Further research should aim to correlate pri-miRNA modulation with fungal viability and host immune responses, optimizing illumination parameters for in vivo translation.

Ultimately, integrating molecular biomarkers such as pri-miRNAs with PDT could pave the way for novel diagnostic–therapeutic approaches in managing fungal infections and inflammation-related oral diseases.

## Figures and Tables

**Table 1:** RT-qPCR primers used to detect the miRNAs by real-time RT-PCR.

Oligo name	Sequence: 5′----------------3′
OG Pri-Mir 146 F	TTTACAGGGCTGGGACAG
OG Pri-Mir 146 R	TCAGGATCTACTCTCTCCAGG
**Oligo name**	**Sequence: 5′-----------------3′**
OG Pri-Mir-155 F	AGGAAGGGGAAATCTGTG
OG Pri-Mir-155 R	TCATGCTTCTTTGTCATCCT

## Data Availability

The original contributions presented in this study are included in the article. Further inquiries can be directed to the corresponding authors.

## References

[1] Taylor M., Brizuela M., Raja A. (2025). Oral Candidiasis 2023.

[2] Hellstein J.W., Marek C.L. (2019). Candidiasis: Red and White Manifestations in the Oral Cavity. Head. Neck Pathol..

[3] Benito-Cruz B., Aranda-Romo S., López-Esqueda F.J., de la Rosa-García E., Rosas-Hernández R., Sánchez-Vargas L.O. (2016). Oral Candida isolates and fluconazole susceptibility patterns in older Mexican women. Arch. Gerontol. Geriatr..

[4] Lorenzo-Pouso A.I., Pérez-Jardón A., Caponio V.C.A., Spirito F., Chamorro-Petronacci C.M., Álvarez-Calderón-Iglesias Ó., Gándara-Vila P., Lo Muzio L., Pérez-Sayáns M. (2022). Oral Chronic Hyperplastic Candidiasis and Its Potential Risk of Malignant Transformation: A Systematic Review and Prevalence Meta-Analysis. J. Fungi.

[5] Zamil D., Rosen T. (2024). Practical Update on Treatment of Oral Candidiasis. J. Drugs Dermatol..

[6] Deng Y.C., Shih C.J., Lin S.Y., Wang L.C., Yang T.Y., Tseng S.P. (2025). Synergistic effect between taurine-induced silver ion and itraconazole against azole-resistant Candida species and *Candida auris*. J. Microbiol. Immunol. Infect..

[7] Contaldo M., Di Stasio D., Romano A., Fiori F., Della Vella F., Rupe C., Lajolo C., Petruzzi M., Serpico R., Lucchese A. (2023). Oral Candidiasis and Novel Therapeutic Strategies: Antifungals, Phytotherapy, Probiotics, and Photodynamic Therapy. Curr. Drug Deliv..

[8] Wu H., Zhang Y., Jiang L., Huang H. (2025). Photodynamic therapy with photodegradable photosensitizers. Chem. Commun..

[9] Haque T., Albagieh H., Akhter F., Alkahwaji A. (2025). Effectiveness of Photodynamic Therapy in the Treatment of Oral Diseases: A Reality or Myth?. Photobiomodul. Photomed. Laser Surg..

[10] Fiegler-Rudol J., Lipka B., Kapłon K., Moś M., Skaba D., Kawczyk-Krupka A., Wiench R. (2025). Evaluating the Efficacy of Rose Bengal as a Photosensitizer in Antimicrobial Photodynamic Therapy Against *Candida albicans*: A Systematic Review. Int. J. Mol. Sci..

[11] Tkaczyk M., Mertas A., Kuśka-Kiełbratowska A., Fiegler-Rudol J., Bobela E., Cisowska M., Morawiec T., Skaba D., Wiench R. (2025). In Vitro Evaluation of *Candida* spp. and *Staphylococcus aureus* Sensitivity to 450 nm Diode Laser-Mediated Antimicrobial Photodynamic Therapy with Curcumin and Riboflavin. Int. J. Mol. Sci..

[12] de Souto Medeiros M.R., da Silva Barros C.C., de Macedo Andrade A.C., de Lima K.C., da Silveira É.J.D. (2023). Antimicrobial photodynamic therapy in the treatment of oral erythematous candidiasis: A controlled and randomized clinical trial. Clin. Oral. Investig..

[13] Casu C., Orrù G. (2024). Potential of photodynamic therapy in the management of infectious oral diseases. World J. Exp. Med..

[14] Kubizna M., Dawiec G., Wiench R. (2024). Efficacy of Curcumin-Mediated Antimicrobial Photodynamic Therapy on *Candida* spp.—A Systematic Review. Int. J. Mol. Sci..

[15] Casu C., Butera A., Piga A., Scribante A., Fais S., Orrù G. (2025). Lactoferrin Solution as a New Natural Photosensitizer in Photodynamic Therapy Against Oral *Candida* spp. Multidrug-Resistant Isolates: A Preliminary In Vitro Study. Microorganisms.

[16] Kordic M., Martinovic D., Puizina E., Bozic J., Zubcic Z., Dediol E. (2024). Impact of Human Papillomavirus on microRNA-21 Expression in Oral and Oropharyngeal Cancer—A Systematic Review. Int. J. Mol. Sci..

[17] Yong X.Z., Zhou Y.X., Wu T.T., Jiang Q.Z., Liu Z.M., Zhang Z.M., He R.Q., Huang Z.G., Chen G., Tao R. (2025). Differential expression of miRNA-769-5p and Smad2 in patients with or without oral cGVHD. Adv. Clin. Exp. Med..

[18] Eslami M., Khazeni S., Khanaghah X.M., Asadi M.H., Ansari M.A., Garjan J.H., Lotfalizadeh M.H., Bayat M., Taghizadieh M., Taghavi S.P. (2023). MiRNA-related metastasis in oral cancer: Moving and shaking. Cancer Cell Int..

[19] Kong X., Diao S., Xu H., Sun J., Ma B. (2022). Association between miRNA-499 gene polymorphism and autoimmune diseases: A meta-analysis. PLoS ONE.

[20] Jin W., Wang J., Liu C.P., Wang H.W., Xu R.M. (2020). Structural Basis for pri-miRNA Recognition by Drosha. Mol. Cell..

[21] Balakittnen J., Ekanayake Weeramange C., Wallace D.F., Duijf P.H.G., Cristino A.S., Hartel G., Barrero R.A., Taheri T., Kenny L., Vasani S. (2024). A novel saliva-based miRNA profile to diagnose and predict oral cancer. Int. J. Oral. Sci..

[22] Hamed M.N., Abdulbaqi H.R. (2024). Expression of miRNAs (146a and 155) in human peri-implant tissue affected by peri-implantitis: A case control study. BMC Oral Health.

[23] Wang L.H., Xu M.L. (2025). Non-invasive diagnosis of pulmonary tuberculosis and predictive potential for treatment outcomes via miR-146a and miR-155 levels. Diagn. Microbiol. Infect. Dis..

[24] Croston T.L., Lemons A.R., Beezhold D.H., Green B.J. (2018). MicroRNA Regulation of Host Immune Responses following Fungal Exposure. Front. Immunol..

[25] Rocha A.R., Inada N.M., da Silva A.P., Bagnato V.S., Buzzá H.H. (2024). Photodynamic inactivation strategies for maximizing antifungal effect against Sporothrix spp. and *Candida albicans* in an in vitro investigation. PLoS Negl. Trop. Dis..

[26] Wiench R., Skaba D., Stefanik N., Kępa M., Gilowski Ł., Cieślar G., Kawczyk-Krupka A. (2019). Assessment of sensitivity of selected Candida strains on antimicrobial photodynamic therapy using diode laser 635 nm and toluidine blue—In vitro research. Photodiagnosis Photodyn. Ther..

[27] Wiench R., Skaba D., Matys J., Grzech-Leśniak K. (2021). Efficacy of Toluidine Blue-Mediated Antimicrobial Photodynamic Therapy on *Candida* spp. A Systematic Review. Antibiotics.

[28] Casu C., Orrù G., Scano A. (2022). Curcumin/H2O2 photodynamically activated: An antimicrobial time-response assessment against an MDR strain of *Candida albicans*. Eur. Rev. Med. Pharmacol. Sci..

[29] Andrade M.C., Ribeiro A.P., Dovigo L.N., Brunetti I.L., Giampaolo E.T., Bagnato V.S., Pavarina A.C. (2013). Effect of different pre-irradiation times on curcumin-mediated photodynamic therapy against planktonic cultures and biofilms of *Candida* spp. *Arch*. Oral Biol..

[30] de Senna A.M., Vieira M.M.F., Machado-de-Sena R.M., Bertolin A.O., Núñez S.C., Ribeiro M.S. (2018). Photodynamic inactivation of Candida ssp. on denture stomatitis. A clinical trial involving palatal mucosa and prosthesis disinfection. Photodiagn. Photodyn. Ther..

[31] Marques Meccatti V., de Souza Moura L., Guerra Pinto J., Ferreira-Strixino J., Abu Hasna A., Alves Figueiredo-Godoi L.M., Campos Junqueira J., Marcucci M.C., de Paula Ramos L., Carvalho C.A.T. (2022). *Curcuma longa* L. Extract and Photodynamic Therapy are Effective against *Candida* spp. and Do Not Show Toxicity In Vivo. Int. J. Dent..

[32] Stepanenko A.A., Dmitrenko V.V. (2015). HEK293 in cell biology and cancer research: Phenotype, karyotype, tumorigenicity, and stress-induced genome-phenotype evolution. Gene.

[33] Perumal P.O., Mhlanga P., Somboro A.M., Amoako D.G., Khumalo H.M., Khan R.M. (2019). Cytoproliferative and Anti-Oxidant Effects Induced by Tannic Acid in Human Embryonic Kidney (Hek-293) Cells. Biomolecules.

[34] Kara M., Boran T., Öztaş E., Jannuzzi A.T., Özden S., Özhan G. (2022). Zoledronic acid-induced oxidative damage and endoplasmic reticulum stress-mediated apoptosis in human embryonic kidney (HEK-293) cells. J. Biochem. Mol. Toxicol..

[35] Arena T.A., Harms P.D., Wong A.W. (2018). High Throughput Transfection of HEK293 Cells for Transient Protein Production. Methods Mol. Biol..

[36] Madhusudana S.N., Sundaramoorthy S., Ullas P.T. (2010). Utility of human embryonic kidney cell line HEK-293 for rapid isolation of fixed and street rabies viruses: Comparison with Neuro-2a and BHK-21 cell lines. Int. J. Infect. Dis..

[37] Schmalz G., Li S., Burkhardt R., Rinke S., Krause F., Haak R., Ziebolz D. (2016). MicroRNAs as Salivary Markers for Periodontal Diseases: A New Diagnostic Approach?. BioMed Res. Int..

[38] Nayar G., Gauna A., Chukkapalli S., Velsko I., Kesavalu L., Cha S. (2016). Polymicrobial infection alter inflammatory microRNA in rat salivary glands during periodontal disease. Anaerobe.

[39] Mascitti M., Orsini G., Tosco V., Monterubbianesi R., Balercia A., Putignano A., Procaccini M., Santarelli A. (2018). An Overview on Current Non-invasive Diagnostic Devices in Oral Oncology. Front. Physiol..

[40] Zhou X., Ying X., Wu L., Liu L., Wang Y., He Y., Han M. (2024). Research Progress of Natural Product Photosensitizers in Photodynamic Therapy. Planta Med..

[41] Pardo A., Butera A., Giordano A., Gallo S., Pascadopoli M., Scribante A., Albanese M. (2023). Photodynamic Therapy in Non-Surgical Treatment of Periodontitis: A Systematic Review and Meta-Analysis. Appl. Sci..

[42] Nie S., Gong Y., Wang A., Guo R., Chen X., Yuan Y. (2025). *Fusobacterium nucleatum* Infection Drives Glutathione Depletion in Gastric Cancer: Integrated Multi-Omics and Experimental Validation. Microorganisms.

[43] Borgia F., Custurone P., Peterle L., Pioggia G., Guarneri F., Gangemi S. (2021). Involvement of microRNAs as a Response to Phototherapy and Photodynamic Therapy: A Literature Review. Antioxidants.

[44] Soonthornchai W., Chaiyapechara S., Jarayabhand P., Söderhäll K., Jiravanichpaisal P. (2015). Interaction of *Vibrio* spp. with the Inner Surface of the Digestive Tract of *Penaeus monodon*. PLoS ONE.

[45] Wei T.T., Cheng Z., Hu Z.D., Zhou L., Zhong R.Q. (2019). Upregulated miR-155 inhibits inflammatory response induced by *C. albicans* in human monocytes derived dendritic cells via targeting p65 and BCL-10. Ann. Transl. Med..

[46] Incognito D., Ciappina G., Gelsomino C., Picone A., Consolo P., Scano A., Franchina T., Maurea N., Quagliariello V., Berretta S. (2025). Fusobacterium Nucleatum in Colorectal Cancer: Relationship Among Immune Modulation, Potential Biomarkers and Therapeutic Implications. Int. J. Mol. Sci..

[47] Hitzler S.U.J., Fernández-Fernández C., Günther K., Dietschmann A., Hovhannisyan H., Möslinger A., Austermeier S., Cristóvão B., Vascelli G., Zelante T. (2025). Host albumin redirects *Candida albicans* metabolism to engage an alternative pathogenicity pathway. Nat. Commun..

[48] Chen T., Zhang Q., Pan J., Xu J., Liu Y., Al-Shroofy M., Cheng Y.T. (2016). Low-Temperature Treated Lignin as Both Binder and Conductive Additive for Silicon Nanoparticle Composite Electrodes in Lithium-Ion Batteries. ACS Appl. Mater. Interfaces.

[49] Casu C., Natoli V., Inchingolo A.M., Orrù G. (2025). Refractory angular cheilitis treated with photodynamic therapy (PDT): An observational pilot study. Photodiagnosis Photodyn. Ther..

[50] Wang Y., Scheiber M.N., Neumann C., Calin G.A., Zhou D. (2011). MicroRNA regulation of ionizing radiation-induced premature senescence. Int. J. Radiat. Oncol. Biol. Phys..

[51] Dhahbi J.M., Atamna H., Boffelli D., Magis W., Spindler S.R., Martin D.I. (2011). Deep sequencing reveals novel microRNAs and regulation of microRNA expression during cell senescence. PLoS ONE.

